# Use of Capture–Recapture to Estimate Underreporting of Ebola Virus Disease, Montserrado County, Liberia

**DOI:** 10.3201/eid2112.150756

**Published:** 2015-12

**Authors:** Etienne Gignoux, Rachel Idowu, Luke Bawo, Lindis Hurum, Armand Sprecher, Mathieu Bastard, Klaudia Porten

**Affiliations:** Epicentre, Paris, France (E. Gignoux, M. Bastard, K. Porten);; Centers for Disease Control and Prevention, Atlanta, Georgia, USA (R. Idowu);; Ministry of Health and Social Welfare, Monrovia, Liberia (L. Bawo);; Médecins Sans Frontières, Brussels, Belgium (L. Hurum, A. Sprecher)

**Keywords:** Ebola, Ebola virus disease, disease outbreaks, disease notification, epidemiologic monitoring, epidemiology, data interpretation, statistical methods, viruses, Liberia

**To the Editor:** Underreporting of cases during a large outbreak of disease is not without precedent (*1*–*5*). Health systems in West Africa were ill-prepared for the arrival of Ebola virus disease (Ebola) (*6*). The Ebola outbreak in Liberia was declared on March 31, 2014, and peaked in September 2014. However, by mid-June, the outbreak had reached Montserrado County, where the capital, Monrovia, is located. In response, the Liberia Ministry of Health and Social Welfare (MOHSW) created a National Ebola Hotline: upon receipt of a call, a MOHSW case investigation team was dispatched to the site of the possible case. Additionally, persons could seek care at an Ebola Treatment Unit (ETU) or be referred to an ETU by another health care facility. During June 1–August 14, 2014, MOHSW, Médecins Sans Frontières, and the US nongovernment organization Samaritan’s Purse managed 3 ETUs in Montserrado County, including 2 in Monrovia operated by Eternal Love Winning Africa (ELWA).

In August 2014, to assess the extent of underreporting in the midst of the Ebola outbreak, we analyzed 2 sources of data collected during June 1–August 14. The first comprised data collected by MOHSW case investigation teams. These data were collected on MOHSW case forms and entered into a database emulating these forms using Epi Info version 7 software (Centers for Disease Control and Prevention, Atlanta, GA, USA). The second data source (designed on Excel 2003; Microsoft, Redmond, WA, USA) comprised data on all patients admitted to the 2 ELWA ETUs (ELWA1 and ELWA2). We used a capture–recapture (CRC) approach.

CRC can evaluate the completeness of reporting and thereby be used to correct for underreporting (*7*). CRC methods use data from overlapping databases to estimate the number of unreported cases and thus more closely derive the true number of Ebola cases. Both databases were populated and managed separately, although the included Ebola cases are assumed to reflect the same patient population in Montserrado County. These 2 databases enabled us to use CRC to estimate the true number of Ebola cases in Montserrado County.

To be included in either database, a case must have been classified as suspected, probable, or confirmed Ebola. The case definitions, following the official MOHSW definition for Ebola, were identical in both databases. Eventually, after laboratory confirmation, cases could be reclassified as “not a case” and thus be excluded from the analysis.

To estimate the total number of Ebola cases during the study period, we used Chapman’s 2-sample CRC population estimate (*7*); we calculated the 95% CI as proposed by Wittes et al. (*8*). We performed a sensitivity analysis measuring impact of error in matching cases during record linkage.

A total of 227 Ebola cases were recorded in the MOHSW database and 99 Ebola cases in the Montserrado County ETUs database (Table). Of these, 25 were found in both databases, 202 in the MOHSW database only, and 74 in the Montserrado County ETU database only. We estimated that the cumulative number of Ebola cases for Montserrado County during the study period was 876 (95% CI 608–1,143).

A sensitivity analysis performed with ±5 cases showed that, with 5 additional cases in common between databases, the cumulative number of cases would decrease to 734 (95% CI 537–931); with 5 additional discordant cases, the estimate would increase to 1,085 (95% CI 700–1,469). Our analysis shows that the number of cases in Montserrado Country was at least 3-fold higher than that reported during the study period.

Our study had several limitations. According to the doctor in charge of data collection up to August 4, some forms (<10) completed at the beginning of June 2014 might have been misplaced. Additionally, some patients who entered the ETU were not recorded in the registry book (˂5). CRC assumes a closed population. In Montserrado County, persons can move freely. In both databases, we included only cases that occurred in or were reported in Montserrado County.

CRC assumes that links between the 2 sources based on identifying case information are error free. The sensitivity analysis suggested that even if up to 5 case matches were not detected, our conclusion was relatively robust.

CRC assumes homogeneity in the likelihood of being captured and recaptured and that data sources are independent. In our analysis, homogeneity is unlikely. For example, the MOHSW database was more likely to capture cases in persons more likely to seek care; the ETU database was more likely to detect cases in persons referred by health workers. Similar behaviors might have resulted in positive dependency in each data source. Both heterogeneity and positive dependency with data sources leads to underestimation.

Despite these limitations, we estimated more Ebola cases than were reported through official channels during the beginning of the outbreak in Montserrado County. Routine studies similar to ours can rapidly provide public health officials managing the outbreak response with estimates of underreporting and enable timely mobilization of appropriate resources. However, we believe that further exploration of this technique to better understand the possible difference of capture preference of each source may help improve the technique and benefit future outbreaks.
